# Objective Assessment of Chronic Pain in Horses Using the Horse Chronic Pain Scale (HCPS): A Scale-Construction Study

**DOI:** 10.3390/ani11061826

**Published:** 2021-06-18

**Authors:** Johannes P. A. M. van Loon, Lucia Macri

**Affiliations:** 1 Department of Equine Sciences, Faculty of Veterinary Medicine, Utrecht University, 3584 CM Utrecht, The Netherlands; 2 Equine Department, Vetsuisse Faculty, University of Zürich, CH-8057 Zürich, Switzerland; lucia.macri@uzh.ch

**Keywords:** chronic, composite, horse facial expression, pain, equine, welfare

## Abstract

**Simple Summary:**

The objective assessment of acute pain in horses can be performed by means of different types of pain scales. To date, no studies have been conducted to develop a structured pain measuring tool for the assessment of chronic pain in horses. In donkeys, a pain scale incorporating behavioural and facial expression-based parameters has been shown to be of added value in assessing chronic pain. In this study, we used the same concept to develop a pain scale (the Horse Chronic Pain Scale; HCPS) to measure chronic pain in horses, based on behavioural parameters and facial expressions. This pain scale was tested in 26 horses with different types of chronic pain (osteoarthritis, chronic laminitis, chronic back and neck problems, chronic dental disorders) and 27 healthy control animals. The authors found the HCPS to be a reproducible pain scale and useful to assess chronic pain in horses. Further studies are needed to confirm these findings in other horses and to validate its use in subsets of horses with specific chronic pain states.

**Abstract:**

The objective assessment of chronic pain is of utmost importance for improving welfare and quality of life in horses. Freedom from disease and pain is one of the ‘five freedoms’ that are necessary for animal welfare. The aim of this study was to develop a pain scale for the assessment of chronic pain in horses (Horse Chronic Pain Scale; HCPS), which is based on behavioural and facial expressions. The scale was used to assess 53 horses (26 horses diagnosed with chronic painful conditions by means of clinical examination and additional diagnostic procedures (consisting of osteoarthritis, chronic laminitis, chronic back and neck problems, chronic dental disorders) and 27 healthy control animals). Animals were assessed once daily for three consecutive days by two observers that were blinded to the condition of the animals and were unaware of any analgesic treatment regimens. The HCPS consists of two parts, the Horse Chronic Pain Composite Pain Scale (HCP CPS, with behavioural parameters) and the EQUUS-FAP (Equine Utrecht University Scale for Facial Assessment of Pain). The HCP CPS had good inter-observer reliability (intraclass correlation coefficient (ICC) = 0.84, *p* < 0.001), while the EQUUS-FAP component (with facial expression-based parameters) had poor inter-observer reliability (ICC = 0.45, *p* < 0.05). The inter-observer reliability of the combined HCPS was good (ICC = 0.78, *p* < 0.001). The HCPS revealed significant differences between horses with chronic painful conditions and control horses on 2 out of 3 days (*p* < 0.05). In conclusion, we tested a composite pain scale for the assessment of chronic pain in horses based on behavioural and facial expression-based parameters. Further studies are needed to validate this pain scale before it can be used in practice.

## 1. Introduction

Welfare and quality of life assessment in horses is receiving growing attention [1,2,3]. Welfare assessment in horses by means of the AWIN protocol (Animal Welfare INdicators) has been described and further evaluated [4,5]. It contains various parameters, including facial expressions of pain (Horse Grimace Scale [6]), but has not been validated for chronic pain. Being free from disease and pain is one of the essential aspects of good welfare; other important aspects are proper feeding and housing conditions and the possibility to express social behaviours. Objective assessment of acute pain in equids has been studied extensively in the last few decades [7,8,9], and both composite and facial expression-based pain scales have proven useful in horses with different types of acute pain [6,10]. Pain assessment has also been described in ridden horses [11] and in horses during tacking-up and mounting [12]. Moreover, in donkeys, a recent study has shown that composite and facial expression-based pain scales can be successfully used [13]. Most studies so far have focused on acute pain assessment, but, recently, chronic pain assessment in donkeys has also been described [14]. Chronic pain is defined as pain that persists after normal healing time and that lasts or recurs for over 3–6 months [15,16]. Chronic pain, especially when gradually evolved, can be difficult to assess, yet it may greatly influence equine welfare. The aim of this study was to develop a composite pain scale for the assessment of chronic pain in horses, including behavioural parameters and facial expressions. Criterion validity and clinical applicability were investigated by assessment of a cohort of horses with chronic painful conditions and healthy pain-free control animals. The purpose of the current study was to perform the first steps in developing a valid pain scale that can be used to assess chronic pain in horses. Further validating steps are needed in future follow-up studies. The hypothesis was that the Horse Chronic Pain Scale (HCPS) can differentiate between horses with and without chronic pain and can be used for repeatable and objective chronic pain assessment in horses.

## 2. Materials and Methods

The institutional Ethics Committee on the Care and Use of Experimental Animals approved the study design in compliance with Dutch and English legislation on animal experimentation. Because the procedures used in this study only consisted of behavioural observations and physiologic assessments (heart rate, breathing rate, borborygmi, rectal temperature) that are routinely taken in the clinical setting and are deemed not likely to cause pain, suffering, distress or lasting harm equivalent to, or higher than, that caused by the introduction of a needle (article 1.5f EU directive 2010/63/EU), ethical approval was granted without a formal application and hence no official approval number was given. Written consent was obtained from owners for all animals participating in this study.

Fifty-three geriatric horses (26 with chronic painful conditions and 27 healthy control animals) that were located at a retirement home for elderly horses in the Netherlands were used in this study. The attending veterinarians and retirement home staff selected horses that met the following inclusion criteria for study enrolment: animals had to be diagnosed with chronically painful health problems (present for more than 3 months) by means of general clinical and lameness examination and additional diagnostic procedures such as radiography or ultrasound by the attending veterinarian, where appropriate. The chronic conditions consisted of 4 horses with chronic laminitis, 9 horses with osteoarthritis, 6 horses with chronic back problems, 5 horses with Equine Odontoclastic Tooth Resorption and Hypercementosis (EOTRH) and 2 horses with other chronic dental problems; these conditions were considered mild by the attending veterinarian and did not require analgesic treatment. The control group was selected based on the absence of a history of chronic disease and no abnormalities in recent daily inspection by the grooms and weekly clinical examination by the attending veterinarian were observed. All control horses remained pain-free during the study period. The horses were all under veterinary supervision and none of them received analgesic treatment. All horses were kept in groups; most of them were turned out during the whole study period, while some of them were stabled during part of the day and then turned out. All assessments were performed while the horses were being turned out; the assessments were performed during daytime and were not related to additional feeding. Assessments lasted for around 15 minutes in total. Our study was a non-interventional study and the pain scale that we have described has been constructed to be tested in the current study. Therefore, at the time of data acquisition, we did not know whether increased scores on the constructed pain scale did indeed indicate that the horses could be experiencing chronic pain and it was chosen not to provide analgesic treatment based on the findings of our study, but to only assess horses during the follow-up period. Analgesic treatment was only decided on the clinical judgement of the attending veterinarian (and was not performed during our study). Table 1 shows the demographic data on the horses that were included in the study. All assessments were performed by the same observer (veterinary research masters student LM), who was blinded to the condition of the animals. A second observer (diplomate in veterinary anaesthesia and analgesia JvL) scored 66.7% of all animals simultaneously with the first observer for inter-observer agreement analysis; these 2 observer assessments were randomly distributed over the dataset. Horses were observed in their home environment on three consecutive days using the Horse Chronic Pain Composite Pain Scale (HCP CPS; Table 2) and the Equine Utrecht University Scale for Facial Assessment of Pain (EQUUS-FAP; Table 3). The order of assessments was always the same: assessments were started with the EQUUS-FAP; then, the observational parameters from HCP CPS were performed. Finally, the remaining non-observational parameters from HCP CPS were assessed. The HCP CPS was developed from the composite pain scale for the assessment of chronic pain in donkeys [14] and was tested in a pilot study with 10 healthy horses that were not included in the current study (van Loon et al., unpublished results). This testing was performed to train the observers that were participating in the current study. The EQUUS-FAP was earlier described in horses with acute pain [17]. The combination of these two scales (scores from both scales were added) resulted in the Horse Chronic Pain Scale (HCPS), which was also analysed separately. Fifty-eight percent of all horses (15 patients and 16 controls) were again assessed during a second period of 3 days in autumn (first observations were during summer). 

All data except for age were expressed as medians and interquartile ranges. Age of both groups was tested for normality with the Shapiro–Wilk test. Differences in mean age of patients and control animals were tested with independent samples t-test. Inter-observer reliability was assessed by means of intraclass correlation (ICC) analysis. ICC values less than 0.5 are indicative of poor reliability, values between 0.5 and 0.75 indicate moderate reliability, values between 0.75 and 0.9 indicate good reliability and values greater than 0.9 indicate excellent reliability [18]. Differences between the 3 consecutive pain assessments were analysed by Friedman tests for patients and control animals separately. Because of significant differences (*p* < 0.05) over these 3 days for horses with painful chronic conditions in the EQUUS-FAP and HCPS scores, pain scores were analysed for these 3 days separately. Differences in pain scores between horses with chronically painful conditions and control horses were tested using Mann–Whitney U tests for all 3 assessment days separately. Differences between summer and autumn assessments for patients and controls were assessed by means of Wilcoxon signed rank tests. Statistical analyses were performed using commercially available software (SPSS statistics version 20.0, IBM, Amsterdam, the Netherlands). Statistical significance was accepted at *p* < 0.05.

## 3. Results

The age of patients and control animals was normally distributed (*p* = 0.11 for patients and *p* = 0.57 for control animals) and was not significantly different (*p* = 0.2). 

### 3.1. Inter-Observer Reliability

**Figure 1:**
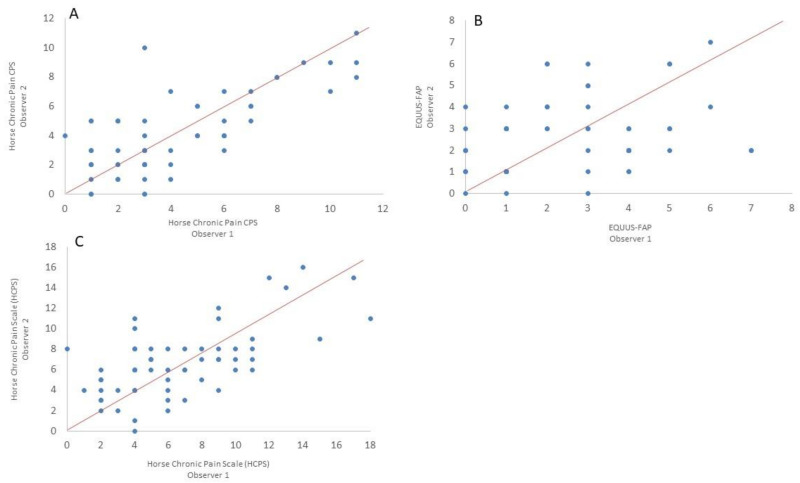
Inter-observer reliability. (**A**) Horse Chronic Pain Composite Pain Scale (HCP CPS), intraclass correlation coefficient (ICC) = 0.84 (confidence interval CI: 0.74–0.91, *p* < 0.001). (**B**) Equine Utrecht University Scale for Facial Assessment of Pain (EQUUS-FAP), ICC = 0.45 (CI: 0.058–0.67, *p* < 0.05). (**C**) Horse Chronic Pain Scale (HCPS; combination of HCP CPS and EQUUS-FAP), ICC = 0.78, (CI: 0.62–0.87, *p* < 0.001). Solid red lines show y = x, (*n* = 53).

The Horse Chronic Pain CPS (HCP CPS) had a good inter-observer correlation (ICC = 0.84, Confidence Interval (CI): 0.74–0.91) that was statistically significant (*p* < 0.001) (Figure 1A). In contrast, EQUUS-FAP had a statistically significant poor inter-observer correlation (ICC = 0.45 (CI: 0.058–0.67, *p* < 0.05, Figure 1B), while the combined Horse Chronic Pain Scale (HCPS) had good agreement between observers (ICC = 0.78, CI: 0.62–0.87) that was statistically significant (*p* < 0.001, Figure 1C). 

### 3.2. Differences Between Horses with Chronic Pain and Healthy Control Animals

**Figure 2:**
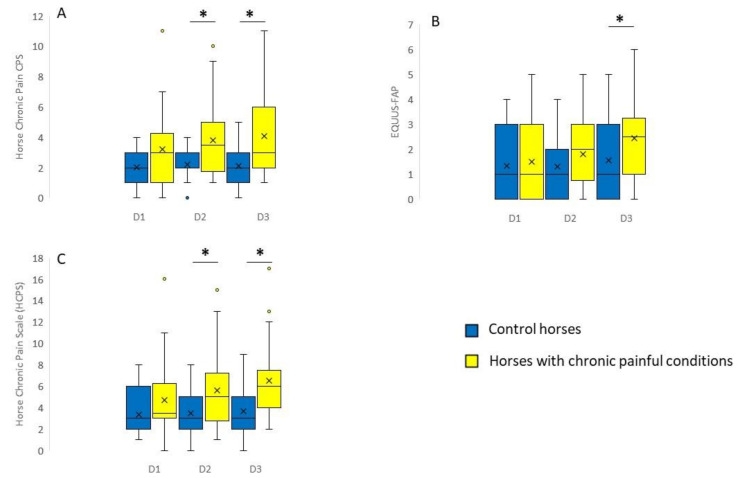
Composite and facial expression-based pain scales in horses with chronically painful conditions and healthy control animals. (**A**) Horse Chronic Pain Composite Pain Scale (HCP CPS). (**B**) Equine Utrecht University Scale for Facial Assessment of Pain (EQUUS-FAP). (**C**) Horse Chronic Pain Scale (HCPS; combination of HCP CPS and EQUUS-FAP). D1, Day 1; D2, Day 2; D3, Day 3. Boxes show 25–75 percentiles, with the horizontal line being the median and the x being the mean score; whiskers show 5–95 percentiles; open dots show outliers. * *p* < 0.05 (*n* = 27 control animals; *n* = 26 horses with chronic painful conditions).

Horses with chronically painful conditions had significantly higher pain scores compared to control animals on two out of three consecutive days for the HCP CPS (*p* < 0.05, Figure 2A) and the combined HCPS (*p* < 0.05, Figure 2C). EQUUS-FAP showed a significant difference only at the third day of evaluation (Figure 2B; *p* < 0.05 for all significant differences). 

### 3.3. Seasonal Variation

**Figure 3:**
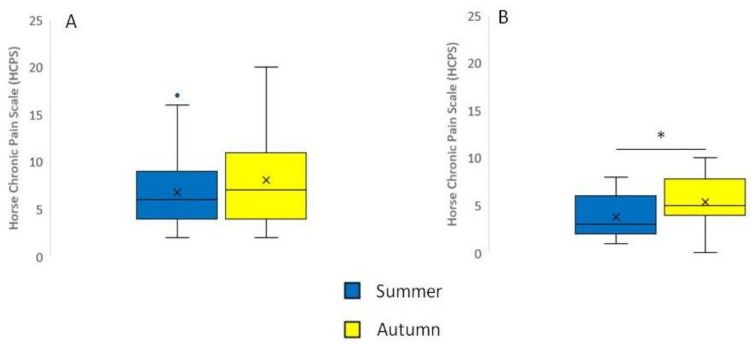
Patients and control animals during summer and autumn observations. (**A**) horses with chronic painful conditions during summer and autumn observations (*p* = 0.20, *n* = 15). (**B**) healthy pain-free control horses during summer and autumn observations. Boxes show 25–75 percentiles, with the horizontal line being the median and the x being the mean score; whiskers show 5–95 percentiles; open dots show outliers (* *p* < 0.05, *n* = 16).

Horses with chronically painful conditions did not have significant differences between pain scores that were assessed during summer and during autumn (*p* = 0.20, Figure 3A). Healthy control horses had a slight and statistically significant increase in pain scores from summer to autumn (*p* < 0.05, Figure 3B). More details of the individual pain scores for all animals included in this study can be found in Supplementary Materials Tables S1 and S2.

## 4. Discussion

The current study shows that the HCPS, with or without the parameters regarding facial expressions, could be clinically applied to assess chronically painful conditions in geriatric horses with mild chronic conditions. 

The most obvious differences between horses with and without chronically painful conditions were found in the composite pain scale parameters. In accordance with the results of a chronic pain scale for donkeys [14], facial expressions proved less sensitive for chronic pain and only showed differences between the two groups on the last day of observations, maybe due to the fact that observers managed to more closely observe the horses. Theoretically, the horses were more acclimatised to the observers being close-by on the third day of observations, leading to them showing more true facial expressions. Torcivia and McDonnell [19] described the influence of a caretaker that was approaching horses suffering from orthopaedic pain; the influence of humans disrupted ongoing discomfort behaviour in these patients. Hypothetically, the facial expressions that the horses in the current study showed might have been influenced by the presence of the observers, decreasing the scores. Gleerup et al. [20] also assessed the influence of an observer on the facial expressions of horses with acute pain and found less pronounced expressions when the horses tried to interact with the observer. In a publication by Dalla Costa et al. [21], the influence of other emotions on facial expressions in horses was described, and they found increased scores for backwards positioning of the ears and strained chewing muscles under stress conditions in the horses. Therefore, other emotional states, induced by the presence of the observers, may also have influenced the facial expressions in the horses of the current study. In a recent publication [22], the effect of stressors such as transportation or social isolation of horses on facial expressions was described as well. Another possible explanation could be that the relatively mild chronic conditions in the patient group may have led to minimal increases in facial expressions of pain. Inter-observer agreement for facial expressions was weak, which might be due to the fact that both observers did not evaluate facial expressions at exactly the same time points. Although only briefly trained, the agreement between the Masters student and the specialist observer for the composite pain scale was good. Therefore, poorer agreement for EQUUS-FAP here could also indicate that the recognition of facial expressions requires more training than composite pain scale assessment does.

The composite pain parameters revealed mildly increased scores for patients compared to control animals, with significant differences found at two out of the three assessment time points. These differences were not as pronounced as found in a study that used a similar pain score in donkeys with chronically painful conditions [14], where higher scores for donkeys with chronic pain conditions were reported. This discrepancy matches the fact that the donkeys were diagnosed with mild to moderate chronic conditions, with several donkeys requiring continued analgesic treatment. The horses with chronic conditions in the current study were diagnosed with mild chronic conditions that did not require daily analgesic treatment. This also reflects the management and welfare vision of the retirement home where this study was conducted, as it upholds the principle that horses should be able to live and do well while in their daily care without analgesic treatment. Horses with chronic conditions had similar pain scores during summer and autumn, while the healthy control horses had slightly but significantly increased pain scores in autumn compared to summer. No horses from any of the two groups changed groups during the study; the increase in the control horses could possibly be explained by the changed weather conditions, such as ambient temperature or humidity. Although the control horses were deemed free from chronic pain, they were elderly horses that were retired and living in a resting house, possibly making them prone to subclinical problems that had not been diagnosed yet. The possible influence of weather conditions on chronic pain has been studied in humans [23,24]. 

One important limitation of the current study was that the study population was drawn from a retirement home for elderly horses. These horses were all geriatric and were not ridden or used for work or leisure anymore. Our study population does therefore not reflect a cross-section of the entire equine population and we cannot easily extrapolate our findings to non-geriatric horses that are used for (ridden) work and leisure. Furthermore, the conditions that the horses in our study population suffered from were relatively mild. Further follow-up studies are therefore necessary to explore whether the currently constructed pain scale can also be used for the valid and repeatable assessment of chronic pain in non-geriatric mature horses with moderate to severe chronically painful diseases. Another limitation of our study was the fact that assessments were performed based on a 15-minute observational period. Although this could lead to a greater risk of missing important signals of chronic pain compared to 24-hour observational studies, the aim of this study was to develop a pain scale that can be used in clinical practice and therefore needs to be feasible in terms of time to complete. A final limitation of our study was the fact that the definitions of our parameters in the pain scale and the use of the terms mild, moderate and obvious or severe that are used in our pain scale are not explicitly defined and are subject to the interpretation of the observer. This leaves the observer with a certain level of subjectivity. We performed an inter-observer reliability analysis to assess the possible effect of these definitions that are used in our pain scales.

Another complementary technique for the objective assessment of chronic pain in horses would be to design and validate a questionnaire for owners. Such questionnaires have been described and validated for assessing responses to treatment in dogs and cats with osteoarthritis [25,26], and this approach has been followed by the authors for owner assessment of equine chronic osteoarthritic pain (Howard et al., unpublished results). Further validation of this tool is currently underway (Morris Animal Foundation grant D21EQ508) and will possibly help to make further improvements in chronic pain assessment. Quality of life assessment in human geriatric medicine is well established and is merely performed by questionnaires alone [27], which are considered the gold standard. However, these questionnaires are merely performed based on the self-assessment and self-report of the patient. Although this is impossible in veterinary medicine, the owners of horses very often have considerable knowledge of their animal’s history and normal daily activities, which makes the information that can be acquired by means of owner questionnaires very valid for the assessment of quality of life, especially when combined with individual animal-targeted clinical assessments. The possibility of potential bias in questionnaire-based studies will need to be taken into account. Behavioural assessment of behaviours such as aggression could also be studied to assess possible signs of chronic pain, as has been described in humans [28] and in horses with chronic pain problems that reacted aggressively to humans [29]. 

The welfare indicators that have been described in the AWIN protocol [4] focus on nutrition, housing conditions, the possibility to express normal behaviour and good health. Absence of physical injuries, disease and pain are essential for good health. In their study, Dalla Costa et al. [4] indicate that pain assessment based on facial expressions (they incorporated the Horse Grimace Scale, that has been described for assessment of postoperative pain after surgical castration [6] and in horses with acute laminitis [30]) could be included and stated that other pain-related behavioural parameters should be investigated to assess both acute and chronic types of pain. The current study shows that the Horse Chronic Pain Scale might be useful for the valid assessment of chronic pain in horses. The use of composite and facial expression-based pain scales such as the current scale, and other pain scales that have been described for different types of acute pain in horses, might help in performing objective and valid assessments of acute and chronic pain in horses. Therefore, welfare assessment could be further optimised by the use of these validated pain scales to assess whether horses are free from acute or chronic pain.

## 5. Conclusions

The findings that are described in the current study indicate that the Horse Chronic Pain Scale (HCPS) might be useful to assess horses with chronically painful conditions. In follow-up studies, newly enrolled horses with or without chronic pain will need to be evaluated, to further refine and validate the current pain scale and to investigate its utility. In these follow-up studies, non-geriatric horses that are used for ridden work could also be included to assess whether the HCPS could be used for the valid assessment of chronic pain in these horses.

## Figures and Tables

**Table 1:** Data of the horses that were included in the study (*n* = 53).

	Patients	Controls
Total number	26	27
Mares	15	14
Geldings	11	13
Warmbloods	17	9
Thoroughbreds	2	0
Draft Horses	5	8
Ponies	2	9
Trotters	0	1
Mean age (S.D.)	28.2 (2.8)	27.2 (3.4)

S.D. = standard deviation. Age was not significantly different between patients and control animals (*p* = 0.2).

**Table 2:** Horse Chronic Pain CPS (Composite Pain Scale).

**1. General Appearance**	**Score**	**8. Body Condition Score (BCS, Scale 1–5, Derived from Brooke Hospital)**	
Alert and/or is interacting with mate/group	0	Normal BCS (3/5)	0
Mildly depressed and/or restless and/or decreased interaction with group mate/group	1	Increased or decreased BCS (2/5 or 4/5)	1
Moderately depressed and/or aggressive or no reaction mate/group	2		
Severely depressed (not responding to very clear and obvious signals like movement or sound)	3	Severely increased or decreased BCS (1/5 or 5/5)	3
**2. Body posture**	**Score**	**9. Muscles (epaxial, gluteal, hamstring and cervical muscles)**	**Score**
Quietly standing and/or one hind leg resting	0	Symmetric muscles, no muscle loss	0
Slightly tucked up abdomen	1	Mild muscle loss	1
Extremely tucked up abdomen and/or hunched back and/or stretching limbs/body and/or mild muscle tremors	2	Moderate muscle loss	2
Extremely tucked up abdomen and/or hunched back and/or stretching limbs/body and extreme muscle tremors	3	Obvious (a)symmetric muscle loss	3
**3. Weight distribution**	**Score**	**10. Reaction to observer(s)**	**Score**
Normal weight distribution (including resting a hindlimb)	0	Reaction to observer(s) and ear movements towards observer	0
Less weight on one leg and/or body displaced slightly backwards	1	Mild decreased reaction or ear movements to observer(s)	1
Less weight on one leg, with only the tip on the ground	2	Moderate decreased reaction or ear movements to observer(s)	2
One leg obviously lifted and/or body obviously displaced backwards	3	No reaction or ear movements to observer(s)	3
**4. Weight shifting of front and hind limbs**	**Score**	**11. Pressure sores on skin**	**Score**
Not seen	0	No pressure sores on skin	0
Mild weight shifting,	1	Mild pressure sores on skin	1
Moderate weight shifting	2	Moderate pressure sores on skin	2
Severe weight shifting	3	Severe pressure sores on skin	3
**5. Head carriage**	**Score**	**12. Pain reaction to palpation of the back**	**Score**
Ear base above withers or eats/drinks (from the ground)	0	No reaction to deep palpation	0
Ear base at level of the withers	1	Mild reaction to superficial palpation	1
Ear base below the withers	2	Moderate reaction to superficial palpation	2
Nose to the ground (not eating)	3	Severe reaction to superficial palpation (ears backward, tends to bite)	3
**6. Eating (present food)**	**Score**	**13. Pain reaction to standardised flexion of front and hind limbs** (performed by picking up the limb and bringing it gently to flexion of fetlock, carpal/tarsal joints and knee or shoulder/elbow joints.)	**Score**
Interested and eats normally or fast	0	No reaction to standardised flexion of limbs	0
Reluctant to take food, but eats normally	1	Mild reaction to standardised flexion of limbs	1
Reluctant to take food and drops the food	2	Moderate reaction to standardised flexion of limbs	2
Not interested in food	3	Severe reaction to standardised flexion of limbs	3
**7. Changes in behaviour to partner/group**	**Score**	**14. Carrot/apple test**	**Score**
Horse is in the group	0	Normal biting and eating of carrot/apple	0
Horse is not in the group, but with his/her mate	1		
		Reluctant to or difficulties with eating carrot/apple	2
Partner/group leaves or has left patient (excluding herd behaviour)	3	Does not want to eat the carrot/apple	3
		**15. Movement**	**Score**
		No reluctance to move and normal gait	0
		Mildly abnormal gait (1 or 2 out of 5 lameness AAEP scale *) and/or stiff walk, not reluctant	1
		Reluctance to walk when motivated and/or severely abnormal gait (3 to 5 out of 5 lameness AAEP scale)	2
		Does not want to walk or is lying down	3
**Total Composite Pain Score**			**/45**

* AAEP scale: 0: Lameness not perceptible under any circumstances. 1: Lameness is difficult to observe and is not consistently apparent, regardless of circumstances (e.g., weight-carrying, circling, inclines, hard surface, etc.). 2: Lameness is difficult to observe at a walk or when trotting in a straight line but consistently apparent under certain circumstances (e.g., weight-carrying, circling, inclines, hard surface, etc.). 3: Lameness is consistently observable at a trot under all circumstances 4: Lameness is obvious at a walk. 5: Lameness produces minimal weight bearing in motion and/or at rest or a complete inability to move. Reference: www.aaep.org (accessed on 17 June 2021).

**Table 3:** Equine Utrecht University Scale for Facial Assessment of Pain (EQUUS-FAP). Assessments were based on a 2-minute observation period, as described in a previous study [17].

Facial Expression	Description	Score
Head	Normal movement Less/no or more/exaggerated movement	0 2
Eyelids	Opened More opened eyes or tightening of eyelids with partial closure Obviously more opened eyes (possibly with a rim of the upper eyelid) or obvious tightening of eyelids with (near) complete closure	0 1 2
Focus	Focused on environment Less focused on environment Not focused on environment	0 1 2
Nostrils	Relaxed A bit more opened, nostrils lifted, wrinkles seen Obviously more opened, nostril flaring, possibly audible breathing	0 1 2
Corners mouth/lips	Relaxed Lifted	0 2
Muscle tone head	No fasciculations Mild fasciculations Obvious fasciculations	0 1 2
Flehming/yawning/smacking of the lips	Not seen Seen	0 2
Teeth grinding and/or moaning	Absent Present	0 2
Ear response to auditory stimulus (clicking sound)	Clear response with both ears or ear closest to source Delayed/reduced response to sounds No response to sounds and / or abnormal ear position	0 1 2
Total		/24

## Data Availability

Data is contained within the article or Supplementary Material.

## Supplementary Materials

The following files are available online at https://www.mdpi.com/article/10.3390/ani11061826/s1, Table S1: FAP scores of horses from both groups, Table S2: CPS scores of horses from both groups.
