# Supplementary material for: Objective Assessment of Chronic Pain in Horses Using the Horse Chronic Pain Scale (HCPS): A Scale-Construction Study
Source: Animals (Basel). 2021 Jun 18;11(6):1826. doi: 10.3390/ani11061826 (PMC8234780; doi:10.3390/ani11061826)
Supplement: Supplementary file 1 [file animals-11-01826-s001.zip › Supplementary material File S1.pdf]

FAP week 1

**Horse**	**Day**	**Time**	**Head**	**Eyelids**	**Focus**	**Nostrils**	**Corners m /l**	**Muscle ton**
1	1		0	0	0	0	0	0
2	1		0	0	0	0	0	0
3	1		0	1	0	0	2	0
4	1		0	0	0	0	0	0
5	1		0	0	0	0	0	0
6	1		0	0	0	0	0	0
7	1		0	0	0	0	0	0
8	1		0	1	0	1	0	0
9	1		0	0	0	0	2	0
10	1		0	0	0	0	0	0
11	1		2	0	0	0	0	0
12	1		0	0	0	0	0	0
1	2		0	0	0	0	0	0
2	2		0	0	0	0	2	0
3	2		0	1	0	0	2	0
4	2		0	0	0	0	2	0
5	2		0	1	0	1	2	0
6	2		0	0	0	0	0	0
7	2		0	0	0	1	2	0
8	2		0	1	0	1	2	0
9	2		0	0	0	0	0	0
10	2		0	0	0	0	2	0
11	2		0	0	0	0	0	0
12	2		0	0	0	0	0	0
1	3		0	1	0	0	0	0
2	3		0	1	0	0	2	0
3	3		0	1	0	0	2	0
4	3		0	0	0	0	0	0
5	3		0	1	0	1	2	0
6	3		0	1	0	1	2	0
7	3		0	0	0	1	2	0
8	3		0	1	0	1	2	0
9	3		0	0	0	0	0	0
10	3		0	0	0	0	0	0
11	3		0	0	0	0	0	0
12	3		0	0	0	1	0	0

FAP week 2

**Horse**	**Day**	**Time**	**Head**	**Eyelids**	**Focus**	**Nostrils**	**Corners m /l**	**Muscle ton**
1	1		0	0	0	0	0	0
2	1		0	0	0	1	0	0
3	1		0	1	0	0	0	0
4	1		0	0	0	0	0	0
5	1		0	1	0	0	0	0
6	1		0	1	0	1	2	0
7	1		0	0	0	0	0	0

8	1		0	0	0	0	0	0
9	1		0	1	0	1	2	0
10	1		0	0	0	0	0	0
11	1		0	0	0	0	0	0
12	1		0	0	0	0	0	0
13	1		0	1	0	0	0	0
14	1		0	0	0	0	0	0
15	1		0	0	0	0	0	0
16	1		0	1	0	0	0	0
c	2		0	0	0	0	0	0
2	2		0	1	0	1	2	0
3	2		0	1	0	0	0	0
4	2		0	0	0	0	0	0
5	2		0	1	0	1	0	0
6	2		0	1	0	1	0	0
7	2		0	1	0	0	0	0
8	2		0	0	0	0	0	0
9	2		0	1	0	1	0	0
10	2		0	1	0	0	0	0
11	2		0	0	0	0	0	0
12	2		0	1	0	0	0	0
13	2		0	1	0	0	0	0
14	2		0	0	0	0	0	0
15	2		0	1	0	1	0	0
16	2		0	1	0	1	0	0
1	3		0	0	0	0	0	0
2	3		0	1	0	0	0	0
3	3		0	1	0	0	0	0
4	3		0	1	0	0	0	0
5	3		0	1	0	1	0	0
6	3		0	1	0	1	2	0
7	3		0	1	0	0	0	0
8	3		0	0	0	0	0	0
9	3		0	1	0	0	0	0
10	3		0	1	0	0	0	0
11	3		0	0	0	0	0	0
12	3		0	1	0	0	0	0
13	3		0	1	0	0	0	0
14	3		0	0	0	0	0	0
15	3		0	1	0	1	0	0
16	3		0	1	0	1	2	0

FAP week 3

Horse	Day	Time	Head	Eyelids	Focus	Nostrils	Corners m /l	Muscle ton
1	1		0	1	0	0	0	0
2	1		0	0	0	0	2	0
3	1		0	0	0	0	0	0

4	1		0	0	0	0	2	0
5	1		0	0	0	0	0	0
6	1		0	1	0	0	0	0
7	1		0	1	0	0	2	0
8	1		0	0	0	0	0	0
9	1		0	0	0	0	0	0
10	1		0	1	0	0	2	0
11	1		0	1	0	0	0	0
12	1		0	0	0	0	0	0
13	1		0	1	0	0	2	0
14	1		0	0	0	0	2	0
1	2		0	1	0	0	0	0
2	2		0	0	0	0	2	0
3	2		0	1	0	0	0	0
4	2		0	1	0	0	0	0
5	2		0	0	0	0	0	0
6	2		0	1	0	0	0	0
7	2		0	1	0	0	2	0
8	2		0	0	0	0	0	0
9	2		0	0	0	0	0	0
10	2		0	1	0	1	2	0
11	2		0	0	0	0	0	0
12	2		0	1	0	0	0	0
13	2		0	1	0	0	0	0
14	2		0	0	0	0	0	0
1	3		0	2	0	1	0	0
2	3		0	2	0	1	2	0
3	3		0	1	0	0	0	0
4	3		0	1	0	0	0	0
5	3		0	0	0	0	0	0
6	3		0	1	0	0	2	0
7	3		0	1	0	0	2	0
8	3		0	1	0	0	0	0
9	3		0	0	0	0	0	0
10	3		0	1	0	0	2	0
11	3		0	0	0	0	0	0
12	3		0	1	0	0	2	0
13	3		0	1	0	0	2	0
14	3		0	1	0	0	0	0

FAP week 4

Horse	Day	Time	Head	Eyelids	Focus	Nostrils	Corners m /l	Muscle ton
1	1		0	1	0	1	2	0
2	1		0	2	0	0	2	0
3	1		0	2	0	1	2	0
4	1		0	0	0	0	0	0
5	1		0	0	0	1	0	0

6	1		0	2	0	0	2	0
7	1		0	1	0	0	0	0
8	1		0	1	0	0	2	0
9	1		0	1	0	0	2	0
10	1		0	1	0	1	0	0
11	1		0	0	0	0	0	0
12	1		0	1	0	2	2	0
1	2		0	1	0	1	2	0
2	2		0	2	0	0	2	0
3	2		0	2	0	1	2	0
4	2		0	0	0	0	0	0
5	2		0	0	0	1	0	0
6	2		0	2	0	0	2	0
7	2		0	1	0	0	0	0
8	2		0	0	0	0	0	0
9	2		0	1	0	0	2	0
10	2		0	1	0	1	0	0
11	2		0	0	0	0	0	0
12	2		0	1	0	2	2	0
1	3		0	1	0	1	2	0
2	3		0	2	0	1	2	0
3	3		0	2	0	1	2	0
4	3		0	1	0	0	0	0
5	3		0	0	0	1	0	0
6	3		0	2	0	0	2	0
7	3		0	1	0	0	0	0
8	3		0	1	0	0	2	0
9	3		0	1	0	0	2	0
10	3		0	1	0	1	0	0
11	3		0	1	0	1	0	0
12	3		0	2	0	2	2	0

FAP week 1 autumn

**Horse**	**Day**	**Time**	**Head**	**Eyelids**	**Focus**	**Nostrils**	**Corners m /l**	**Muscle ton**
1	1		0	0	0	0	0	0
2	1		0	1	0	0	2	0
3	1		0	2	0	2	0	0
4	1		0	2	0	2	2	0
5	1		0	0	0	0	0	0
6	1		0	1	0	0	2	0
7	1		0	0	0	0	0	0
8	1		0	1	0	0	0	0
9	1		0	0	0	0	0	0
10	1		0	1	0	0	0	0
11	1		0	0	0	0	0	0
12	1		0	0	0	0	0	0
13	1		0	0	0	0	0	0

14	1		0	0	0	1	0	0
15	1		0	1	0	0	0	0
16	1		0	0	0	0	0	0
1	2		0	0	0	1	0	0
2	2		0	1	0	0	2	0
3	2		0	2	0	2	2	0
4	2		0	1	0	2	2	0
5	2		0	0	0	0	0	0
6	2		0	1	0	0	2	0
7	2		0	1	0	0	0	0
8	2		0	1	0	0	2	0
9	2		0	0	0	0	0	0
10	2		0	1	0	0	2	0
11	2		0	0	0	0	0	0
12	2		0	0	0	0	0	0
13	2		0	1	0	0	2	0
14	2		0	0	0	1	0	0
15	2		0	1	0	0	2	0
16	2		0	0	0	1	0	0
1	3		0	0	0	1	0	0
2	3		0	1	0	1	2	0
3	3		0	2	0	2	2	0
4	3		0	1	0	2	2	0
5	3		0	0	0	0	0	0
6	3		0	1	0	1	2	0
7	3		0	1	0	1	0	0
8	3		0	1	0	0	2	0
9	3		0	1	0	1	2	0
10	3		0	2	0	0	2	0
11	3		0	0	0	0	0	0
12	3		0	0	0	0	0	0
13	3		0	1	0	1	2	0
14	3		0	0	0	1	0	0
15	3		0	1	0	0	2	0
16	3		0	0	0	1	0	0

FAP week 2 autumn

Horse	Day	Time	Head	Eyelids	Focus	Nostrils	Corners m /l	Muscle ton
1	1		0	0	0	1	0	0
2	1		0	0	0	0	0	0
3	1		0	2	0	1	2	0
4	1		0	1	0	0	0	0
5	1		0	1	0	1	2	0
6	1		0	1	0	1	0	0
7	1		0	1	0	0	0	0
8	1		0	1	0	1	2	0
9	1		0	2	0	0	2	0

10	1	0	2	0	0	2	0
11	1	0	0	0	0	0	0
12	1	0	1	0	0	0	0
13	1	0	1	0	0	0	0
14	1	0	1	0	0	0	0
15	1	0	1	0	1	0	0
16	1	0	1	0	1	2	0
1	2	0	0	0	1	0	0
2	2	0	1	0	0	0	0
3	2	0	2	0	1	2	0
4	2	0	1	0	0	0	0
5	2	0	2	0	1	2	0
6	2	0	1	0	1	0	0
7	2	0	2	0	0	0	0
8	2	0	1	0	1	0	0
9	2	0	2	0	1	2	0
10	2	0	2	0	0	2	0
11	2	0	0	0	0	0	0
12	2	0	0	0	1	0	0
13	2	0	0	0	0	0	0
14	2	0	1	1	0	0	0
15	2	0	1	0	1	0	0
16	2	0	1	0	1	2	0
1	3	0	0	0	1	0	0
2	3	0	1	0	0	0	0
3	3	0	2	0	1	2	0
4	3	0	1	0	0	0	0
5	3	0	2	0	1	2	0
6	3	0	1	0	1	0	0
7	3	0	1	0	0	0	0
8	3	0	1	0	1	0	0
9	3	0	2	0	1	2	0
10	3	0	2	0	2	0	0
11	3	0	0	0	0	0	0
12	3	0	1	0	0	0	0
13	3	0	0	0	0	0	0
14	3	0	1	0	1	0	0
15	3	0	1	0	1	0	0
16	3	0	1	0	1	2	0

Flehming	Teethgrind	Ear response	Totaal
0	0	0	0
0	0	0	0
0	0	0	3
0	0	0	0
0	0	0	0
0	0	0	0
0	0	0	0
0	0	0	2
0	0	0	2
0	0	0	0
0	0	0	2
0	0	0	0
0	0	0	0
0	0	0	2
0	0	0	3
0	0	0	2
0	0	0	4
0	0	0	0
0	0	0	3
0	0	0	4
0	0	0	0
0	0	0	2
0	0	0	0
0	0	0	0
0	0	0	1
0	0	0	3
0	0	0	3
0	0	0	0
0	0	0	4
0	0	0	4
0	0	0	3
0	0	0	4
0	0	0	0
0	0	0	0
0	0	0	0
0	0	0	1

Flehming	Teethgrind	Ear response	Totaal
0	0	0	0
0	0	0	1
0	0	0	1
0	0	0	0
0	0	0	1
0	0	0	4
0	0	0	0

0	0	0	0
0	0	0	4
0	0	0	0
0	0	0	0
0	0	0	0
0	0	0	1
0	0	0	0
0	0	0	0
0	0	0	1
0	0	0	0
0	0	0	4
0	0	0	1
0	0	0	0
0	0	0	2
0	0	0	2
0	0	0	1
0	0	0	0
0	0	0	2
0	0	0	1
0	0	0	0
0	0	0	1
0	0	0	1
0	0	0	0
0	0	0	2
0	0	0	2
0	0	0	0
0	0	0	1
0	0	0	1
0	0	0	1
0	0	0	2
0	0	0	4
0	0	0	1
0	0	0	0
0	0	0	1
0	0	0	1
0	0	0	0
0	0	0	1
0	0	0	1
0	0	0	0
0	0	0	2
0	0		4

**Flehming**	**Teethgrind**	**Ear response**	**Totaal**
0	0	0	1
0	0	0	2
0	0	0	0

0	0	0	2
0	0	0	0
0	0	0	1
0	0	0	3
0	0	0	0
0	0	0	0
0	0	0	3
0	0	0	1
0	0	0	0
0	0	0	3
0	0	0	2

0	0	0	1
0	0	0	2
0	0	0	1
0	0	0	1
0	0	0	0
0	0	0	1
0	0	0	3
0	0	0	0
0	0	0	0
0	0	0	4
0	0	0	0
0	0	0	1
0	0	0	1
0	0	0	0

0	0	0	3
0	0	0	5
0	0	0	1
0	0	0	1
0	0	0	0
0	0	0	3
0	0	0	3
0	0	0	1
0	0	0	0
0	0	0	3
0	0	0	0
0	0	0	3
0	0	0	3
0	0	0	1

**Flehming**	**Teethgrind**	**Ear response**	**Totaal**
0	0	0	4
0	0	0	4
0	0	0	5
0	0	0	0
0	0	0	1

0	0	0	4
0	0	0	1
0	0	0	3
0	0	0	3
0	0	0	2
0	0	0	0
0	0	0	5

0	0	0	4
0	0	0	4
0	0	0	5
0	0	0	0
0	0	0	1
0	0	0	4
0	0	0	1
0	0	0	0
0	0	0	3
0	0	0	2
0	0	0	0
0	0	0	5

0	0	0	4
0	0	0	5
0	0	0	5
0	0	0	1
0	0	0	1
0	0	0	4
0	0	0	1
0	0	0	3
0	0	0	3
0	0	0	2
0	0	0	2
0	0	0	6

**Flehming**	**Teethgrind**	**Ear response**	**Totaal**
0	0	0	0
0	0	0	3
0	0	0	4
0	0	0	6
0	0	0	0
0	0	0	3
0	0	0	0
0	0	0	1
0	0	0	0
0	0	0	1
0	0	0	0
0	0	0	0
0	0	0	0

0	0	0	1
0	0	0	1
0	0	0	0

0	0	0	1
0	0	0	3
0	0	0	6
0	0	0	5
0	0	0	0
0	0	0	3
0	0	0	1
0	0	0	3
0	0	0	0
0	0	0	3
0	0	0	0
0	0	0	0
0	0	0	3
0	0	0	1
0	0	0	3
0	0	0	1

0	0	0	1
0	0	0	4
0	0	0	6
0	0	0	5
0	0	0	0
0	0	0	4
0	0	0	2
0	0	0	3
0	0	0	4
0	0	0	4
0	0	0	0
0	0	0	0
0	0	0	4
0	0	0	1
0	0	0	3
0	0		1

**Flehming**	**Teethgrind**	**Ear response**	**Totaal**
0	0	0	1
0	0	0	0
0	0	0	5
0	0	0	1
0	0	0	4
0	0	0	2
0	0	0	1
0	0	0	4
0	0	0	4

0	0	0	4
0	0	0	0
0	0	0	1
0	0	0	1
0	0	0	1
0	0	0	2
0	0	0	4
0	0	0	1
0	0	0	1
0	0	0	5
0	0	0	1
0	0	0	5
0	0	0	2
0	0	0	2
0	0	0	2
0	0	0	5
0	0	0	4
0	0	0	0
0	0	0	1
0	0	0	0
0	0	1	3
0	0	0	2
0	0	0	4
0	0	0	1
0	0	0	1
0	0	0	5
0	0	0	1
0	0	0	5
0	0	0	2
0	0	0	1
0	0	0	2
0	0	0	5
0	0	0	4
0	0	0	0
0	0	0	1
0	0	0	0
0	0	0	2
0	0	0	2
0	0		4
