# Supplementary material for: Objective Assessment of Chronic Pain in Horses Using the Horse Chronic Pain Scale (HCPS): A Scale-Construction Study
Source: Animals (Basel). 2021 Jun 18;11(6):1826. doi: 10.3390/ani11061826 (PMC8234780; doi:10.3390/ani11061826)
Supplement: Supplementary file 1 [file animals-11-01826-s001.zip › Supplementary material File S2.pdf]

CPS week 1

Horse	Day	Time	1	2	3	4	5	6
1	1		0	0	0	0	0	0
2	1		0	0	0	0	0	0
3	1		0	0	0	0	0	2
4	1		0	0	0	0	0	2
5	1		0	0	0	0	0	0
6	1		0	0	0	0	0	0
7	1		0	0	0	0	0	0
8	1		0	0	0	0	0	0
9	1		0	0	0	0	0	0
10	1		1	0	0	0	0	0
11	1		0	0	0	0	0	0
12	1		0	0	0	0	0	
1	2		0	0	0	0	0	0
2	2		0	0	0	0	0	0
3	2		0	0	0	0	0	2
4	2		0	0	0	0	0	2
5	2		0	0	0	0	0	0
6	2		0	0	0	0	0	2
7	2		0	0	0	0	0	0
8	2		0	0	0	0	0	0
9	2		0	0	0	0	0	0
10	2		1	0	0	0	0	0
11	2		0	0	0	0	0	0
12	2		0	0	0	0	0	0
1	3		0	0	0	0	0	0
2	3		0	0	0	0	0	0
3	3		0	0	0	0	0	2
4	3		0	0	0	0	0	2
5	3		0	0	0	0	0	2
6	3		0	0	0	0	0	0
7	3		0	0	0	0	0	0
8	3		0	0	0	0	0	0
9	3		0	0	0	0	0	0
10	3		1	0	0	0	0	0
11	3		0	0	0	0	0	0
12	3		0	0	0	0	0	0

CPS week 2

Horse	Day	Time	1	2	3	4	5	6
1	1		0	0	0	0	0	0
2	1		0	0	0	0	0	0
3	1		0	0	0	0	0	0
4	1		0	0	0	0	0	0
5	1		0	0	0	0	0	0
6	1		0	0	0	0	0	0
7	1		0	0	0	0	0	0

8	1		0	0	0	0	0	0
9	1		0	0	0	0	0	0
10	1		0	0	0	0	0	0
11	1		0	0	0	0	0	0
12	1		0	0	0	0	0	0
13	1		0	0	0	0	0	0
14	1		0	0	0	0	0	0
15	1		0	0	0	0	0	0
16	1		0	0	0	0	0	0
1	2		0	0	0	0	0	0
2	2		0	0	0	0	0	0
3	2		0	0	0	0	0	0
4	2		0	0	0	0	0	0
5	2		0	0	0	0	0	0
6	2		0	0	0	0	0	0
7.2	2		0	0	0	0	0	0
8	2		0	0	0	0	0	3
9	2		1	0	0	0	1	0
10	2		0	0	0	0	0	0
11	2		0	0	0	0	0	0
12	2		0	0	0	0	0	3
13	2		0	0	0	0	0	0
14	2		0	0	0	0	0	0
15	2		0	0	0	0	0	0
16	2		0	0	0	0	0	0
1	3		0	0	0	0	0	0
2	3		0	0	0	0	0	0
3	3		0	0	0	0	0	0
4	3		0	0	0	0	0	0
5	3		0	0	0	0	0	0
6	3		0	0	0	0	0	0
7.2	3		0	0	0	0	0	0
8	3		0	0	0	0	0	0
9	3		0	0	0	0	0	0
10	3		0	0	0	0	0	0
11	3		0	0	0	0	0	0
12	3		0	0	0	0	0	0
13	3		1	0	0	0	0	0
14	3		0	0	0	0	0	0
15	3		0	0	0	0	0	0
16	3		0	0	0	0	0	0

CPS week 3

**Horse**	**Day**	**Time**	**1**	**2**	**3**	**4**	**5**	**6**
1	1		0	0	0	0	0	0
2	1		0	0	0	0	0	0
3	1		0	0	0	0	0	0

4	1		0	0	0	0	0	0
5	1		0	0	0	0	0	0
6	1		0	0	0	0	0	0
7	1		0	0	0	0	0	0
8	1		0	0	0	0	0	0
9	1		0	0	0	0	0	0
10	1		0	0	0	0	0	0
11	1		0	0	0	0	0	0
12	1		0	0	0	0	0	0
13	1		0	0	0	0	0	0
14	1		0	0	0	0	0	0
1	2		0	0	0	0	0	0
2	2		0	0	1	0	0	0
3	2		0	0	0	0	0	0
4	2		0	0	0	0	0	0
5	2		0	0	0	0	0	0
6	2		0	0	0	0	0	0
7	2		0	0	0	0	0	0
8	2		0	0	0	0	0	0
9	2		0	0	0	0	0	0
10	2		0	0	0	0	0	0
11	2		0	0	0	0	0	0
12	2		0	0	0	0	0	0
13	2		0	0	0	0	0	0
14	2		0	0	0	0	0	0
1	3		0	0	0	0	0	0
2	3		1	0	1	1	0	0
3	3		0	0	0	0	0	0
4	3		0	0	0	0	0	0
5	3		0	0	0	0	0	0
6	3		0	0	0	0	0	0
7	3		0	0	0	0	0	0
8	3		0	0	0	0	0	0
9	3		0	0	0	0	0	0
10	3		0	0	0	0	0	0
11	3		0	0	0	0	0	0
12	3		0	0	0	0	0	0
13	3		0	0	0	0	0	0
14	3		0	0	0	0	0	0

CPS week 4

**Horse**	**Day**	**Time**	**1**	**2**	**3**	**4**	**5**	**6**
1	1		0	0	0	0	0	0
2	1		0	0	0	0	0	0
3	1		0	0	1	1	0	0
4	1		0	0	0	0	0	0

5	1		0	0	0	0	0	0
6	1		0	0	1	0	0	0
7	1		0	0	0	0	0	0
8	1		0	0	0	0	0	0
9	1		0	0	0	0	0	0
10	1		0	0	0	0	0	0
11	1		0	0	0	0	0	0
12	1		0	0	1	2	0	0
1	2		0	0	0	0	0	0
2	2		0	0	0	0	0	0
3	2		0	0	0	0	0	0
4	2		0	0	0	0	0	0
5	2		0	0	0	0	0	0
6	2		0	0	0	0	0	0
7	2		0	0	0	0	0	0
8	2		0	0	0	0	0	0
9	2		0	0	0	0	0	0
10	2		0	0	0	0	0	0
11	2		0	0	0	0	0	0
12	2		0	0	1	2	0	0
1	3		0	0	0	0	0	0
2	3		0	0	0	0	0	0
3	3		0	0	1	1	0	0
4	3		0	0	0	0	0	0
5	3		0	0	0	0	0	0
6	3		0	0	0	0	0	0
7	3		0	0	0	0	0	0
8	3		0	0	0	0	0	0
9	3		0	0	0	0	0	0
10	3		0	0	0	0	0	0
11	3		0	0	0	0	0	0
12	3		1	0	0	0	1	1

CPS week 1 autumn

**Horse**	**Day**	**Time**	**1**	**2**	**3**	**4**	**5**	**6**
1	1		0	0	0	0	0	3
2	1		0	0	0	0	0	0
3	1		0	0	0	0	0	0
4	1		0	0	0	0	0	0
5	1		0	0	0	0	0	0
6	1		0	0	0	0	0	0
7	1		0	0	0	0	0	0
8	1		0	0	0	0	0	0
9	1		0	0	0	0	0	3
10	1		0	0	0	0	0	3
11	1		0	0	0	0	0	0
12	1		0	0	0	0	0	0

13	1		0	0	0	0	0	0
14	1		0	0	0	0	0	0
15	1		0	0	0	0	0	0
16	1		0	0	0	0	0	0
1	2		0	0	0	0	0	0
2	2		0	0	0	0	0	0
3	2		0	0	0	0	0	0
4	2		0	0	0	0	0	0
5	2		0	0	0	0	0	0
6	2		0	0	0	0	1	0
7	2		1	0	0	0	2	0
8	2		0	0	0	0	0	0
9	2		0	0	0	0	0	0
10	2		0	0	0	0	0	0
11	2		0	0	0	0	0	0
12	2		0	0	0	0	0	0
13	2		0	0	0	0	0	0
14	2		0	0	0	0	1	0
15	2		0	0	0	0	0	0
16	2		0	0	0	0	0	0
1	3		0	0	0	0	0	0
2	3		0	0	0	0	0	0
3	3		0	0	0	0	1	0
4	3		0	0	0	0	0	0
5	3		1	0	0	0	0	3
6	3		0	0	0	0	1	0
7	3		0	0	0	0	0	0
8	3		0	0	0	0	0	0
9	3		0	0	0	0	1	3
10	3		0	0	0	0	0	0
11	3		0	0	0	0	2	3
12	3		0	0	0	0	1	0
13	3		0	0	0	0	0	0
14	3		0	0	0	0	1	0
15	3		0	0	0	0	0	0
16	3		0	0	0	0	1	0

CPS week 2 autumn

**Horse**	**Day**	**Time**	**1**	**2**	**3**	**4**	**5**	**6**
1	1		0	0	0	0	0	3
2	1		0	0	0	0	1	3
3	1		1	0	1	0	1	3
4	1		0	0	0	0	0	0
5	1		1	0	0	0	1	0
6	1		0	0	0	0	0	3
7	1		0	0	0	0	0	0
8	1		0	0	0	0	0	0

9	1	0	0	0	0	1	0
10	1	1	0	1	0	1	0
11	1	0	0	0	0	0	0
12	1	0	0	0	0	1	3
13	1	0	0	0	0	0	0
14	1	1	0	0	0	1	3
15	1	0	0	0	0	0	0
16	1	0	0	0	0	0	0
1	2	1	0	0	0	1	3
2	2	1	0	0	0	1	3
3	2	0	0	0	0	0	0
4	2	0	0	0	0	0	0
5	2	0	0	1	0	1	0
6	2	0	0	0	0	0	3
7	2	0	0	0	0	0	0
8	2	1	0	0	0	1	3
9	2	0	0	0	0	0	0
10	2	1	0	1	0	1	0
11	2	0	0	0	0	0	0
12	2	0	0	0	0	1	3
13	2	0	0	1	1	1	3
14	2	1	0	0	0	2	3
15	2	0	0	0	0	0	0
16	2	0	0	0	0	0	0
1	3	0	0	0	0	0	3
2	3	1	0	0	0	1	0
3	3	1	0	1	0	2	3
4	3	0	0	0	0	0	3
5	3	0	0	0	0	1	0
6	3	0	0	0	0	0	0
7	3	0	0	0	0	0	0
8	3	1	0	0	0	1	3
9	3	0	0	0	0	0	0
10	3	1	0	1	0	1	0
11	3	0	0	1	0	0	0
12	3	0	0	0	0	0	3
13	3	0	0	1	1	0	0
14	3	0	0	0	0	0	3
15	3	0	0	0	0	0	0
16	3	0	0	0	0	0	0

**7**	**8**	**9**	**10**	**11**	**12**	**13**	**14**	**15**
0	0	3	0	0	0	0	0	0
0	0	0	0	0	0	0	0	0
0	3	0	1	0	0	0	0	0
0	0	0	0	0	0	0	0	2
0	0	0	0	0	0	1	1	1
0	0	1	0	0	0	0	0	0
0	0	1		0	0	0	1	0
0	2	0	0	0	0	0	0	0
0	1	1	0	0	0	0	0	0
0	0	0	0	0	1	0	0	0
0	3	1	0	0	0	0	0	0
0	0	1	1	0	0	0	1	0
0	0	3	0	0	0	0	0	0
0	2	0	1	0	0	0	0	0
0	3	0	1	0	0	0	0	2
0	2	0	0	0	1	0	0	2
0	3	1	1	0	0	0	3	2
0	0	1	0	0	0	0	0	0
0	3	1	0	0	0	0	2	0
0	3	0	0	0	0	0	0	0
0	0	1	0	0	0	1	0	0
0	2	0	0	0	1	0	0	0
0	0	1	0	0	0	0	0	0
0	0	1	1	0	0	0	1	0
0	1	3	0	0	0	0	0	0
0	3	0	1	0	0	0	0	0
0	3	0	1	0	0	0	0	2
0	0	0	0	0	1	0	0	2
0	3	1	1	0	0	0	3	2
0	3	1	0	0	0	0	0	0
0	3	1	0	0	0	0	1	0
0	0	3	0	0	0	0	0	0
0	0	1	0	0	0	0	0	0
0	0	0	0	0	1	0	0	0
0	0	1	0	0	0	0	0	0
0	1	1	1	0	0	0	1	0

**7**	**8**	**9**	**10**	**11**	**12**	**13**	**14**	**15**
0	0	0	1	0	0	0	0	0
0	1	0	1	0	0	0	1	0
0	1	0	0	0	0	0	1	0
0	0	1	2	0	0	0	1	0
0	1	1	1	0	0	0	3	0
0	3	0	1	0	0	0	0	2
0	0	0	2	0	0	0	0	0

0	0	0	1	0	0	0	0	0
0	3	1	0	0	0	0	2	0
0	0	0	1	0	0	0	0	0
0	0	0	1	0	0	0	1	1
0	0	0	1	0	0	0	1	0
0	1	0	1	0	1	0	0	0
0	0	1	2	0	0	0	0	0
0	0	1	2	0	0	0	0	0
0	1	0	1	0	0	0	0	1

0	0	0	1	0	0	0	0	0
0	3	0	1	0	0	0	0	1
0	1	1	0	0	0	0	0	0
0	0	1	2	0	0	0	1	0
0	2	1	1	0	0	0	3	1
0	2	0	0	0	0	0	0	1
0	1	0	1	0	0	0	0	0
0	0	0	1	0	0	0	0	0
0	2	3	0	0	0	0	2	0
0	1	0	0	0	0	0	0	1
0	0	0	1	0	0	0	1	1
0	1	0	0	0	0	0	0	0
0	1	1	1	0	1	0	0	0
0	0	0	1	2	0	0	0	0
0	2	1	2	0	0	0	0	0
0	2	0	1	0	0	1	0	1

0	0	0	1	0	0	0	0	1
0	1	0	1	0	0	0	0	1
0	1	1	0	0	0	0	2	0
0	1	1	2	0	0	0	1	0
0	2	1	1	0	0	0	3	1
0	3	0	0	0	0	0	0	1
0	1	0	1	0	0	0	0	0
0	0	0	1	0	0	0	0	0
0	1	3	0	0	0	0	2	0
0	1	0	0	0	0	0	0	1
0	0	0	1	0	0	0	0	1
0	1	0	0	0	0	0	0	0
0	1	1	1	0	1	0	0	0
0	0	0	1	2	0	0	0	0
0	2	1	2	0	0	0	0	0
0	3	0	1	0	0	1	0	1

**7**	**8**	**9**	**10**	**11**	**12**	**13**	**14**	**15**
0	3	1	1	0	0	0	0	0
0	2	1	1	0	0	0	0	0
0	0	1	0	0	0	0	0	0

0	2	0	1	0	0	0	0	0
0	0	1	1	0	0	0	0	0
0	1	0	1	0	0	0	0	0
0	3	1	2	0	0	0	0	0
0	0	0	1	0	0	0	0	0
0	0	1	1	0	0	0	0	0
0	3	0	2	0	0	0	2	0
0	1	0	0	0	0	0	0	0
0	0	0	0	0	0	0	0	0
0	3	0	1	0	0	0	0	0
0	2	1	0	0	0	0	0	0

0	1	1	1	0	0	0	0	0
0	2	1	1	0	0	0	1	0
0	1	1	0	0	0	0	0	0
0	1	0	1	0	0	0	1	0
0	0	1	1	0	0	0	0	0
0	1	0	1	0	0	0	0	0
0	3	1	2	0	0	0	0	0
0	0	0	1	0	0	0	0	0
0	0	1	1	0	0	0	0	0
0	3	0	2	0	0	0	2	0
0	0	0	0	0	0	0	0	0
0	1	0	0	0	0	0	1	0
0	1	0	1	0	0	0	0	0
0	0	1	0	0	0	0	0	0

0	3	0	1	0	0	0	0	0
0	3	1	1	0	0	0	1	0
0	1	1	0	0	0	0	0	0
0	1	0	1	0	0	0	0	0
0	0	1	1	0	0	0	0	0
0	3	0	1	0	0	0	0	0
0	3	1	2	0	0	0	0	0
0	1	0	1	0	0	0	0	0
0	0	1	1	0	0	0	0	1
0	3	0	2	0	0	0	2	0
0	0	0	0	0	0	0	0	0
0	3	0	0	0	0	0	1	0
0	3	0	1	0	0	0	0	0
0	1	1	0	0	0	0	1	0

**7**	**8**	**9**	**10**	**11**	**12**	**13**	**14**	**15**
0	3	1	1	0	0	0	1	0
0	3	1	0	0	0	0	0	1
0	3	1	1	0	0	0	1	0
0	0	1	2	0	0	0	0	0

0	1	1	0	0	0	0	0	0
0	3	0	2	0	0	0	0	0
0	1	0	1	0	0	0	0	0
0	3	1	1	0	0	0	0	0
0	3	1	1	0	0	0	1	0
0	2	3	1	0	0	0	0	2
0	0	0	2	0	0	0	0	0
0	3	3	2	0	0	0	1	0

0	3	1	1	0	0	0	1	0
0	3	1	0	0	0	0	0	1
0	3	1	1	0	0	0	0	0
0	0	1	2	0	0	0	0	0
0	1	1	0	0	0	0	0	0
0	3	0	2	0	0	0	0	0
0	1	0	1	0	0	0	0	0
0	0	1	1	0	0	0	0	0
0	3	1	1	0	0	0	1	0
0	2	3	1	0	0	0	0	2
0	0	0	2	0	0	0	0	1
0	3	3	2	0	0	0	0	0

0	3	1	1	0	0	0	1	1
0	3	1	0	0	0	0	0	0
0	3	1	1	0	0	0	1	0
0	1	1	2	0	0	0	0	0
0	1	1	0	0	0	0	0	0
0	3	0	2	0	0	0	0	0
0	1	0	1	0	0	0	0	0
0	3	1	1	0	0	0	0	0
0	3	1	1	0	0	0	0	0
0	2	3	1	0	0	0	0	2
0	2	0	2	0	0	0	0	0
1	3	3	2	0	0	0	1	0

**7**	**8**	**9**	**10**	**11**	**12**	**13**	**14**	**15**
3	0	1	0	0	0	0	0	0
0	3	1	3	0	0	0	0	2
0	3	1	2	0	0	0	3	0
0	3	0	1	0	0	0	0	0
0	0	1	0	0	0	0	2	0
0	3	0	2	0	0	0	0	3
0	0	0	1	0	0	0	0	0
0	1	0	1	0	0	0	0	0
0	0	2	0	0	0	0	0	0
0	1	0	1	0	0	1	1	0
0	0	1	1	0	0	0	0	0
0	0	0	0	0	0	0	0	0

0	0	3	2	0	0	0	0	0
0	1	1	1	0	0	0	0	0
0	1	3	1	0	0	1	0	0
0	0	1	2	0	0	0	0	0

0	1	1	0	0	0	0	0	0
0	3	1	3	0	0	0	1	2
0	3	1	2	0	0	0	3	0
0	3	0	1	0	0	0	0	0
0	0	1	0	0	0	0	3	0
0	3	0	2	0	0	0	0	3
0	1	0	1	0	0	0	1	0
0	3	0	1	0	0	0	0	0
0	0	2	0	0	0	0	0	0
0	3	0	1	0	0	1	1	0
0	0	1	1	0	0	0	0	0
0	0	0	0	0	0	0	0	0
0	3	3	2	0	0	1	1	0
0	1	1	1	0	0	0	0	0
0	3	3	1	0	0	1	0	0
0	1	1	2	0	0	0	0	0

3	1	1	0	0	0	1	0	0
0	2	1	2	0	0	0	1	2
0	3	1	2	0	0	0	3	0
0	3	0	1	0	0	0	0	0
3	0	1	0	0	0	0	3	1
0	3	0	2	0	0	0	0	3
0	2	0	1	0	0	0	0	0
1	3	0	1	0	0	0	0	0
3	3	2	0	0	0	0	0	0
0	3	0	1	0	0	1	1	0
0	0	1	1	0	0	0	0	0
1	0	0	0	0	0	0	0	0
0	3	3	2	0	0	2	2	0
1	1	1	1	0	0	1	1	0
0	3	3	1	0	0	1	0	1
0	1	1	2	0	0	0	1	0

**7**	**8**	**9**	**10**	**11**	**12**	**13**	**14**	**15**
0	1	3	0	0	0	0	1	0
0	0	1	0	0	0	0	0	0
3	3	1	1	0	1	0	3	0
0	1	3	0	0	0	0	0	0
0	3	1	1	0	0	0	0	0
0	2	1	1	0	0	0	0	0
0	1	0	2	0	0	0	0	0
0	3	1	0	0	0	0	0	0

0	3	0	1	0	0	0	1	0
0	3	0	0	0	0	0	0	0
3	0	0	0	0	0	1	0	0
0	1	0	0	0	0	0	1	0
0	1	1	0	0	0	0	0	0
0	1	3	0	0	0	0	1	1
0	2	1	1	0	0	0	0	0
0	3	3	1	0	0	0	0	0

0	1	3	0	0	0	0	2	0
0	1	1	0	0	0	0	0	0
0	3	1	1	0	1	3	3	0
0	1	3	0	0	0	0	0	0
0	3	1	1	0	0	0	1	0
0	2	1	1	0	0	0	0	0
0	2	0	2	0	0	0	0	0
0	3	1	0	0	0	0	0	0
0	3	0	1	0	0	0	1	0
0	3	0	0	0	0	0	1	0
0	0	0	0	0	0	1	0	0
0	1	0	0	0	0	0	1	0
0	0	1	0	0	0	0	0	0
0	3	3	0	0	0	0	1	1
0	2	1	1	0	0	0	1	0
0	3	3	1	0	0	0	1	0

0	1	3	0	0	0	0	2	0
0	1	1	0	0	0	0	0	0
0	3	1	1	0	1	3	3	0
0	1	3	0	0	0	0	0	0
0	3	1	1	0	0	0	1	0
0	2	1	1	0	0	0	1	0
0	0	1	0	2	0	0	0	0
0	2	1	0	0	0	0	0	0
0	3	0	1	0	0	0	1	0
0	3	0	0	0	0	0	0	0
0	0	0	0	0	0	1	0	0
0	1	0	0	0	0	0	1	0
0	0	1	0	0	0	0	1	0
0	2	3	0	0	0	0	1	1
0	2	1	1	0	0	0	1	0
0	3	3	1	0	0	0	1	0

16	Totaal
0	3
1	1
0	6
0	4
0	3
0	1
1	3
1	3
0	2
0	2
0	4
0	3
0	3
1	4
0	8
0	7
2	12
1	4
1	7
1	4
0	2
0	4
0	1
0	3
0	4
1	5
0	8
0	5
1	13
0	4
1	6
1	4
0	1
0	2
0	1
0	4

16	Totaal
0	1
0	3
1	3
0	4
2	8
1	7
2	4

0	1
1	7
0	1
0	3
0	2
0	3
1	4
2	5
0	3
0	1
0	5
1	3
0	4
2	10
1	4
0	2
1	5
1	10
0	2
2	5
1	5
1	5
1	4
2	7
0	5
0	2
0	3
1	5
0	5
2	10
0	4
1	3
1	2
1	7
0	2
1	3
1	2
0	5
1	4
2	7
0	6
**16**	**Totaal**
0	5
0	4
0	1

0	3
0	2
0	2
0	6
0	1
0	2
0	7
0	1
0	0
0	4
0	3
0	3
0	6
0	2
0	3
0	2
0	2
0	6
0	1
0	2
0	7
0	0
1	3
0	2
0	1
0	4
2	11
0	2
0	2
0	2
0	4
0	6
0	2
1	4
1	8
0	0
1	5
0	4
1	4
**16**	**Totaal**
0	6
0	5
1	9
0	3

1	3
1	7
0	2
1	6
1	7
1	9
0	2
2	14
0	6
1	6
1	6
1	4
1	3
1	6
0	2
0	2
1	7
1	9
0	3
2	13
0	7
1	5
1	9
0	4
1	3
1	6
0	2
1	6
1	6
1	9
0	4
2	15

**16**	**Totaal**
0	7
0	9
1	10
1	5
1	4
0	8
0	1
1	3
1	6
0	7
0	2
0	0

1	6
1	4
0	6
0	3
0	2
0	10
2	11
0	4
1	5
0	9
1	7
1	5
0	2
0	6
0	2
0	0
1	11
1	5
0	8
1	5
0	6
1	9
3	13
0	4
1	13
0	9
0	3
1	6
1	13
0	6
0	7
0	2
2	14
1	8
0	9
1	7

**16**	**Totaal**
1	9
0	5
1	19
0	4
0	7
0	7
0	3
0	4

0	6
0	6
0	4
1	7
0	2
0	11
0	4
2	9
1	12
0	7
2	14
0	4
1	9
1	8
0	4
1	10
0	5
1	8
0	1
1	7
1	8
1	15
0	5
1	9
1	10
0	4
2	21
0	7
1	8
1	6
0	3
1	9
0	5
0	6
0	2
1	6
1	5
1	11
0	5
1	9
